# Fluorescence-Tagged Transgenic Lines Reveal Genetic Defects in Pollen Growth—Application to the Eif3 Complex

**DOI:** 10.1371/journal.pone.0017640

**Published:** 2011-03-07

**Authors:** Bijoyita Roy, Gregory P. Copenhaver, Albrecht G. von Arnim

**Affiliations:** 1 Department of Biochemistry, Cellular and Molecular Biology, The University of Tennessee, Knoxville, Tennessee, United States of America; 2 Department of Biology and the Carolina Center for Genome Sciences, The University of North Carolina at Chapel Hill, Chapel Hill, North Carolina, United States of America; 3 Lineberger Comprehensive Cancer Center, The University of North Carolina School of Medicine, Chapel Hill, North Carolina, United States of America; Iowa State University, United States of America

## Abstract

**Background:**

Mutations in several subunits of eukaryotic translation initiation factor 3 (eIF3) cause male transmission defects in *Arabidopsis thaliana*. To identify the stage of pollen development at which eIF3 becomes essential it is desirable to examine viable pollen and distinguish mutant from wild type. To accomplish this we have developed a broadly applicable method to track mutant alleles that are not already tagged by a visible marker gene through the male lineage of Arabidopsis.

**Methodology/Principal Findings:**

Fluorescence tagged lines (FTLs) harbor a transgenic fluorescent protein gene (XFP) expressed by the pollen-specific *LAT52* promoter at a defined chromosomal position. In the existing collection of FTLs there are enough XFP marker genes to track nearly every nuclear gene by virtue of its genetic linkage to a transgenic marker gene. Using FTLs in a *quartet* mutant, which yields mature pollen tetrads, we determined that the pollen transmission defect of the *eif3h-1* allele is due to a combination of reduced pollen germination and reduced pollen tube elongation. We also detected reduced pollen germination for *eif3e*. However, neither *eif3h* nor *eif3e*, unlike other known gametophytic mutations, measurably disrupted the early stages of pollen maturation.

**Conclusion/Significance:**

eIF3h and eIF3e both become essential during pollen germination, a stage of vigorous translation of newly transcribed mRNAs. These data delimit the end of the developmental window during which paternal rescue is still possible. Moreover, the FTL collection of mapped fluorescent protein transgenes represents an attractive resource for elucidating the pollen development phenotypes of any fine-mapped mutation in Arabidopsis.

## Introduction

The genome of flowering plants encodes two life stages that differ dramatically in complexity, anatomy, and interaction with their environment. The diploid sporophyte stage is free-living and metabolically autonomous, while the haploid gametophyte stage depends on support from the parent plant and serves as a carrier of genetic material during sexual reproduction. The male and female gametophytes are referred to as pollen and embryo sacs, respectively. Comparative transcriptome analysis between the sporophyte and the male gametophyte datasets has revealed pollen specific gene expression patterns in higher plants [1], [2]. The large number of pollen specific transcripts observed reflects the striking functional specialization of the male gametophyte.

Compared to transcriptome profiling, very little proteomic analysis has been performed during male gametophyte development, thus limiting our understanding of the critical molecular events [3]–[5]. It has been proposed that many of the mRNA transcripts generated during early pollen maturation, prior to germination, are stored in an untranslated state while the pollen grain desiccates. By comparison, pollen germination and pollen tube growth are thought to be phases of vigorous mRNA translation [6]. Consistent with this model, data from *Nicotiana tabacum* (tobacco) pollen suggest that translationally silenced mRNAs are stored in the form of ribonucleoprotein particles with ribosomal subunits, translation factors and cytoskeletal proteins, and their translation is activated in response to an appropriate trigger such as hydration [7]. The idea of specific translational control during pollen development also gains credence from the identification of pollen specific translation factors [8]–[10] and preferential expression of certain translation factor mRNAs [11].

The gametophyte life stages are amenable to genetic analysis [12], [13]. Determining the genotype of an individual sporophyte is usually straightforward using the polymerase chain reaction or related molecular techniques. However, because the gametophyte generation of flowering plants encompasses only a few cell generations, determining the genotype of individual male gametophytes on a large scale is challenging [14]. One elegant solution for genotyping pollen makes use of a mutant allele that is molecularly tagged with a cell autonomous marker gene, such as β-glucuronidase (GUS) [12] or any fluorescent protein that is expressed in pollen [15]–[17]. However, many mutations that affect the male gametophyte are either not tagged or the marker gene is not expressed in pollen. Therefore the ‘linked-marker’ method has not been broadly applicable.

We reasoned that it would be equally helpful to instead tag the wild-type allele, rather than the mutant allele, given that pollen development is typically examined among the meiotic products of a plant that is heterozygous for one wild-type and one mutant allele. Previously, a large collection of Arabidopsis lines was generated [18]–[20] that each contain a transgene expressing a fluorescent protein (yellow, cyan, or red; XFP collectively) using the post-meiotic, pollen-specific, *LAT52* promoter [21]. Lines with intergenic chromosomal positions were selected after the inserts were physically mapped. These so-called fluorescent tagged lines (FTLs) were made in the *quartet1-2* (*qrt1-2)* mutant background, which sheds mature pollen as meiotically related tetrads [22].

We used two different FTLs to follow the transmission of two mutant alleles of the translation initiation factor eIF3 through male gametophytes. With 12 subunits [23], eIF3 is by far the most complex of the eIFs. It stimulates loading of the 40S subunit of the ribosome with a ternary complex composed of eIF2, tRNA-Met, and GTP and also promotes binding of the 40S ribosomal subunit to the mRNA, in part by connecting with the G subunit of the cap binding complex, eIF4F [24]–[25]. The functions of the individual eIF3 subunits are not well understood. A wild-type copy of eIF3h is necessary for efficient reinitiation on a subset of mRNAs that harbor upstream open reading frames in their 5′ untranslated leader sequences. This function is disrupted in alleles that truncate the carboxyl-terminus of eIF3h [26]–[29]. The h and e subunits of eIF3 form part of the functional core of mammalian eIF3 [30], but neither is a core component of the six-subunit eIF3 complex that exists in budding yeast [24]. Mutations in *eIF3h* reduce male gametophytic transmission [26] while mutation of *eIF3e* eliminates it [31]. Likewise, *eIF3f* is also essential for male transmission [17]. Arabidopsis eIF3e, h, and f are all encoded by single genes. Most *eIF3* subunit genes, including *eIF3e* and *eIF3h*, are highly expressed in pollen. Using *LAT52:XFP* marker genes genetically linked to *eIF3h* and *eIF3e* we defined the developmental stage of the defects induced by their mutant alleles. The mutations did not affect pollen maturation but did hamper pollen germination and pollen tube growth.

## Results

We were interested to determine the stage of pollen development at which individual eIF3 subunits become essential. The possibilities include, immediately after meiosis, during pollen grain maturation, pollen germination, or pollen tube growth. To track and compare the wild-type and mutant *eif3h* alleles we used FTL567, a *LAT52:eYFP* transgene that lies 298 kb (∼1.5 cM) away from the *eIF3h* gene (At1g10840). For *eIF3e*, we used FTL1413, a *LAT52:dsRed2* transgene that lies 1,536 kbp (∼7.7 cM) away from *eIF3e* (At3g57290). As a control, we used FTL800, a *LAT52:DsRed2* transgene on chromosome 2 that is unlinked to either *eIF3h* or *eIF3e*.

**Figure 1:**
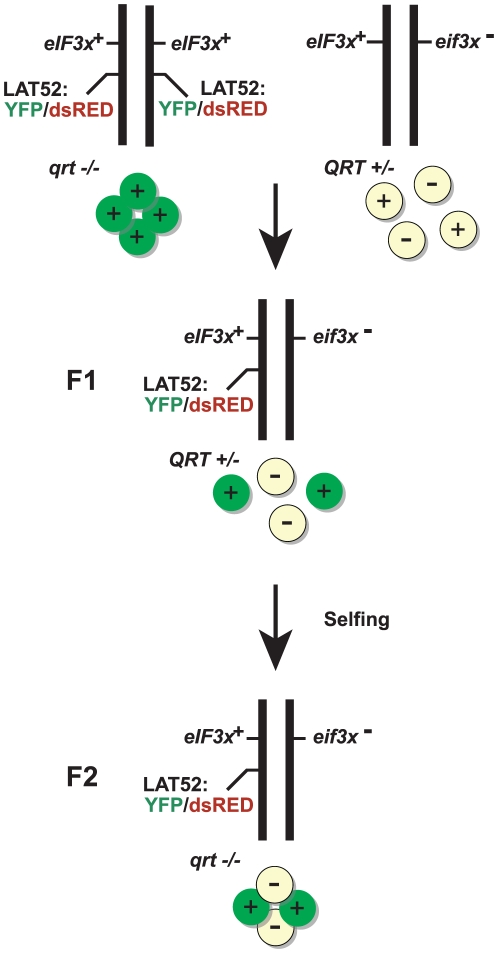
Crossing scheme to generate a fluorescence-tagged line harboring a heterozygous mutation in the *quartet1* (*qrt1*) mutant background. The mutation of interest depicted here is a generic mutation in a subunit of translation initiation factor 3 (*eIF3x*).

**Figure 2:**
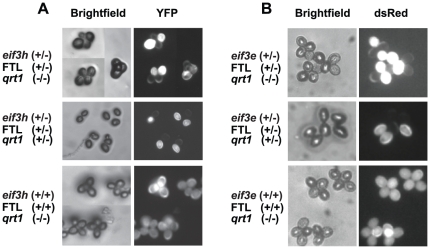
Fluorescence-tagged pollen grains prior to germination. (**A**) Mature *eif3h-1* mutant pollen grains (unmarked) in comparison with wild type (marked with YFP). In the middle panel one mutant pollen grain has germinated prematurely. (**B**) Mature *eif3e-1* mutant pollen grains (unmarked) in comparison with wild-type pollen (marked with DsRed). The genotypes of the parent plants are indicated to the left of each panel.

**Figure 3:**
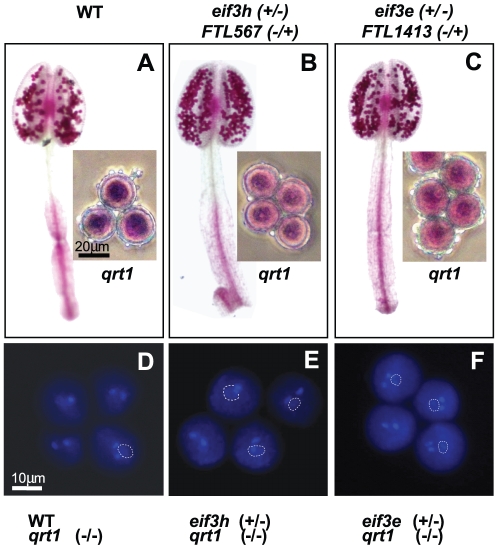
Pollen grains prior to germination. (**A–C**) Mature pollen grains were stained with Alexander's stain to check for viability. (**A**) wild type, (**B**) *eif3h-1*, and (**C**) *eif3e-1* mutant. (**D–F**) Mature pollen tetrads were stained with DAPI to visualize the nuclei. (**D**) wild type, (**E**) *eif3h-1*, and (**F**) *eif3e-1* mutant. The location of the vegetative nucleus (dotted outline) was confirmed by refocusing when it was not in the same plane as the two sperm nuclei. Genotypes of the anther bearing plants are indicated.

Plants harboring the recessive *quartet1* (*qrt1-2*) mutation generate pollen grains that remain joined to each other in meiotic tetrads after anthesis because of an underlying genetic defect in a pectin methylesterase [32]. Plants of the genotype *eif3h^−^/eIF3h^+^; FTL567/+; qrt1-2/qrt1-2* were generated using the crossing scheme shown in **Fig. 1**. As controls, we also selected FTL-tagged plants that were wild-type for *QRT1*. FTL-tagged eIF3h heterozygotes produced pollen tetrads in which two grains were clearly fluorescent (i.e. *eIF3h^+^*) and two were non-fluorescent (*eif3h^−^*) (**Fig. 2A**). Deviations from the 2∶2 segregation were rarely observed, indicating that the *LAT52:eYFP* transgene in FTL567 is expressed reliably and does not become spontaneously silenced. To address the possibility of false negative fluorescence data (i.e. *eIF3h^+^;FTL567* pollen that nevertheless appeared non-fluorescent), we also examined pollen from plants that were homozygous for *eIF3h^+^;FTL567*. In these plants only between 0% and 3% of pollen grains appeared YFP-negative, a low percentage. The four pollen grains from plants segregating *eif3h^−^* and *eIF3h* ^+^ were morphologically indistinguishable, and all four were routinely scored as viable using Alexander's stain (**Fig. 3A, B**). DAPI staining of the wild-type pollen grains revealed one weakly stained vegetative nucleus and two brightly stained sperm nuclei, as expected. The three nuclei reside close to each other in the center of the pollen grain, forming the male germ unit [33]. The same results were obtained for the *eif3h-1* mutant pollen grains (**Fig. 3D, E**). Thus, the *eif3h* mutation causes no dramatic defect during the first stage of post-meiotic pollen development, the stage that encompasses the two subsequent mitotic divisions that result first in one vegetative nucleus and then the two sperm nuclei [34].

**Figure 4:**
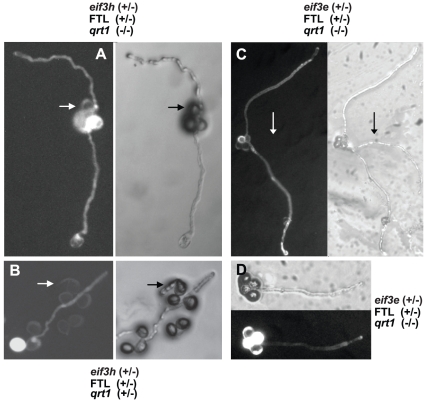
Germination of *eif3h* and *eif3e* mutant pollen. (**A, B**) While pollen germination of *eif3h-1* pollen (non-fluorescent) was low (**A,** also see **Table 1**), such pollen grains did occasionally germinate (**B**, arrow). (**C, D**) Similar results were obtained for *eIF3e*. The tetrad shown (**C**) clearly demonstrates that at least one of two *eif3e-1* mutant pollen has germinated. Generally, however, *eif3e-1* pollen germinates poorly, as seen in the representative tetrad below (**D**). Note that, in the long exposures needed to visualize fluorescence from pollen tubes, the bright fluorescence of the marked pollen grains often reflects from the cell wall of unmarked pollen grains.

**Figure 5:**
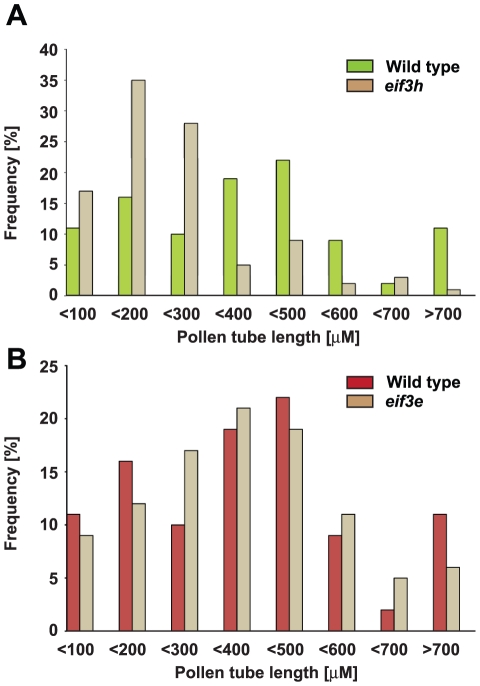
Pollen tube lengths in *eif3h* and *eif3e* mutants. Pollen tube lengths were measured for one hundred non-fluorescent germinated pollen grains of (**A**) *eif3h-1* and (**B**) *eif3e-1* and compared with one hundred fluorescent (*bona fide* wild-type) pollen tubes. Data from two independent replicates were pooled. The difference in mean length was significant for *eif3h* (P<10^−6^, t-test), but not for *eif3e* (P>0.05).

When pollen from *eif3h* ^−^/*eIF3h^+^;FTL567/+* heterozygotes was germinated in pollen germination medium, the non-fluorescent (i.e. *eif3h* mutant) pollen germinated at a much reduced frequency compared to fluorescent (i.e. *eIF3h^+^* wild-type pollen (**Fig. 4A, B** **,** **Table 1**). Moreover, the mutant pollen tubes were shorter than wild type (**Fig. 5A**). However, no significant aberration in pollen tube morphology (bifurcated pollen tubes, bulbous tip, etc.) was observed. Taken together, these analyses indicate that the developmental defect of the *eif3h* mutant male gametophyte occurs as a result of reduced germination frequency (∼1/4 of wild type) and reduced pollen tube growth (∼1/2 of wild type). We did not examine *eif3h* pollen growth in the natural environment of the pistil's transmitting tract. However, reciprocal crosses between *eif3h* and wild type showed a reduced transmission of the *eif3h* ^−^ mutant allele (2.6% [14]). Considering the poor transmission and the in vitro pollen growth defects described here, we surmise that *eif3h* pollen growth is compromised in the pistil as well.

**Table 1:** Germination of pollen from *eIF3h^+^; FTL567/eif3h^−^; +* heterozygous parents.

	*Genotype*		*Total pollen grains*			*Germinated*		
Plant	*FTL567*	*QRT1*	*FL+*	*FL-*	*% FL+*	*FL+*	*FL-*	*%FL+*
1	+/−	+/ .	122	109	53	84	17	83
2	+/−	+/ .	138	163	46	91	31	75
3	+/−	+/ .	145	145	50	64	12	84
4	+/−	−/−	119	94	56	56	13	81
**Average 1–4**	+/−				**51**			**81**
5	FTL800 +/−	−/−	112	119	48	95	93	51

Results from four plants are shown. The map distance between *eIF3h^+^* and *FTL567* is ∼1.5 cM. Germinating pollen tubes were biased toward *eIF3h^+^*; for each plant, the deviation from a 1∶1 segregation was significant at P<10^−6^ by Chi-square test. In contrast, the ‘Total’ ratios did not deviate from 1∶1 (P>0.05). Plants producing tetrads (#4, *qrt1/qrt1)* behaved identical to plants that did not. Results with an *FTL* transgene that is not linked to *eIF3h* are given for comparison (*FTL800*).

Data from FTL-tagged *eIF3e* resembled those for *eIF3h* insofar as we detected no defects in meiosis, pollen grain morphology, viability, or number of nuclei (**Fig. 2B** **,** **Fig. 3C, F**). As in the case of the *eif3h* mutant, the germination of the non-fluorescent (predominantly *eif3e* mutant) pollen grains was reduced compared to wild type (**Fig. 4C, D** **,** **Table 2**). However, unlike *eif3h*, the pollen tubes that did germinate had a normal length distribution (**Fig. 5B**). This cannot be explained by crossing over in the 7.7 cM interval between *eIF3e* and the *LAT52:DsRed2* gene in FTL1413, because there were more than 8% non-fluorescent pollen tubes. Because we have never observed transmission of the *eif3e* mutation through the male gametophyte [31], it appears that the mutation must cause an additional defect, downstream from pollen tube growth. This defect appears to occur prior to fertilization of female gametophytes, because *eif3e* ^−^ */eIF3e^+^* heterozygous plants generate fruits with no apparent abortion of unfertilized or embryo-defective ovules (not shown). In summary, these data indicate that FTLs are a useful resource for delineating the consequences of genetic defects in individual steps of male gametophyte development.

**Table 2:** Germination of pollen from *eIF3e^+^; FTL1413/eif3e* ^−^ *; +* heterozygous parents.

	*Genotype*		*Total pollen grains*			*Germinated*		
Plant	*FTL*	*QRT1*	*FL+*	*FL-*	*% FL+*	*FL+*	*FL-*	*%FL+*
1	+/−	+/ .	92	86	52	57	21	73
2	+/−	+/ .	34	48	41	24	11	69
3	+/−	+/ .	67	61	52	43	17	72
4	+/−	+/ .	59	67	47	37	16	70
5	+/−	+/ .	41	39	51	25	9	74
**Average 1–5**	+/−				**49**			**72**

These plants did not produce tetrads (*QRT1* ^+^). Among the germinated pollen tubes, a clear bias towards FL+ is evident (only 28% were FL-). The deviation from a 1∶1 segregation was significant at P<0.05 for each plant (Chi-square test). The ‘Total’ ratios did not deviate from 1∶1 (P>0.05). Based on the map distance between *eIF3e* and *FTL1413* (7.7 cM) only about 8% of FL- pollen grains are expected to be eIF3e^+^. Therefore, it appears that *eif3e^−^* mutant pollen grains did germinate at a low percentage.

## Discussion

We found that the *eif3h* and *eif3e* mutations examined here did not measurably disrupt early pollen maturation. Instead, their mutant defects became most apparent during pollen germination and, in the case of *eif3h*, pollen tube growth. The timing of these defects is noteworthy given that many other gametophytic mutations (the *pollen development defective* class of mutants*; pdd*) disrupt pollen maturation at a stage prior to germination [12]. While the timing of the two *eif3* mutant phenotypes might be governed in part by paternal rescue, the results are also consistent with the notion that pollen germination and pollen tube growth require vigorous translation of new mRNAs.

In the present study we explored the merit of transgenic lines that express a fluorescent protein from a pollen-specific promoter for the purpose of tracking specific mutations through the male gametophytic lineage of Arabidopsis. The linked XFP transgene allows one to assign wild-type and mutant genotypes, with a high degree of confidence, to individual pollen grains, using simple fluorescence microscopy. This technique offers advantages compared to alternatives. The first alternative, a plant that generates only mutant pollen because it is homozygous for the mutation, may be neither available nor desirable, because it would then be impossible to distinguish sporophytic from gametophytic functions of the gene. Second, in a heterozygote without the XFP marker gene, it is not possible to confidently assign wild-type and mutant genotypes to pollen, even given the 2∶2 segregation in the *qrt1-2* tetrads, because pollen germination and pollen tube growth can be quite variable, even in the wild type. Third, the perfect solution, an XFP transgene that is inserted into the gene of interest itself is most likely not available. Finally, the FTL technique is broadly applicable. A collection of 98 lines with mapped single-T-DNA *LAT52:XFP* insertions is available [20] (**Table S1**).

There are a number of considerations when using the FTL-tagging technique. For example, it relies on consistent expression of the XFP, i.e. absence of spontaneous gene silencing. The FTLs were prescreened for single-locus T-DNAs, which should minimize complications from epigenetic silencing [18]. Nevertheless, checking for gene silencing, which would appear as a fraction of tetrads that have fewer than two fluorescent pollen grains is recommended; normal variability in the success of pollen development also results in a small fraction of non-fluorescent grains.

The potential also exists that the insertion of the *LAT52:XFP* transgene might itself cause a male gametophytic defect even though the insertions have been selected on the basis of occurring in annotated intergenic regions [18]. It is straightforward to control for this, using the XFP heterozygous, but otherwise wild-type siblings of the F1 plant shown in **Fig. 1**.

An obvious disadvantage to the system is that the genetic linkage between the gene of interest and the XFP transgene is in most cases incomplete. As a result, a predictable percentage of fluorescent pollen will be mis-scored. For example, canonical meiotic crossover frequencies would predict that 1.5% of *eif3h* pollen were mis-scored as wild-type and *vice versa*. We were mindful of this circumstance when interpreting results. Here, again, the *quartet1* mutation helps to guard against misinterpretation. In the *quartet1* background, only 3% of meiotic tetrads will contain one pair (one *eif3h* and one *eIF3h+*) of mis-scored pollen grains, while the remaining 97% of tetrads will be marked correctly (the very small percentage of tetrads with double crossover should be negligible in most cases). For this reason, a conclusion that is supported by the great majority of tetrads (100 - 2x[map distance]%) should not be confounded by incomplete linkage. In addition, among the tetrads that have one crossover in the gene-FTL interval, one pair of pollen grains will still be marked correctly. Therefore, finding tetrads where both unmarked grains show an unexpected phenotype should carry a lot of weight and should prompt one to reexamine the conclusion. Another drawback of marking the wild-type allele instead of the mutant allele is that it is more difficult to discern defects in late stages of pollen development. However, with additional effort, it is possible to identify crossover progeny in which the mutant allele is now linked to, and thus positively identified by, the XFP locus. A final consideration to keep in mind while using the system is that while the *quartet1* mutation can be beneficial, it can also potentially interfere with the results, for example, if the phenotype of the mutant of interest was enhanced or suppressed by *qrt1*. For this reason it is recommended to confirm whether the gametophytic phenotypes from tetrad-bearing plants match those from *QRT1/qrt1* heterozygous plants, which do not generate tetrads. The latter can be easily identified from the pedigree in **Fig. 1**. Examining multiple sibling plants to check for consistent results is also advisable to rule out ecotype effects when the mutation of interest is not in the Columbia ecotype.

The pedigree recommended in **Fig. 1** works well for mutations that have a closely linked *XFP* gene, and for mutations that cannot be made homozygous or that are difficult to trace because of a lack of phenotype or lack of a marker gene. In this pedigree, the repulsion between FTL and the mutation of interest helps to select the desired genotype from the F2. Ideally, one simply has to screen the F2 plants for tetrads and select those with tetrads segregating 2∶2 for the FTL locus. Barring a rare crossover, these plants are *mut/MUT; FTL/+*. An alternate strategy is to first cross the mutation of interest to the *quartet1* background, and then cross suitable *mut/MUT; qrt1/QRT* progeny to *FTL/+; qrt1/qrt1.* This strategy does not exploit the repulsion between the mutation and FTL, but might be more suitable if the mutation is initially homozygous, and if it is easy to identify *mut* heterozygotes.

## Materials and Methods

### Plant material

The *eif3h-1* allele (At1g10840) is in the Wassiliweskija (WS) ecotype and harbors a T-DNA insertion in the 10th of 12 exons [26]. The *eif3e-1* allele (At3g57290) is in the Columbia ecotype and harbors T-DNA insertion SALK_121004 in the third of 8 exons [31]. *eif3h-1* harbors an active kanamycin resistance gene that could be used for selection of heterozygous carriers. In crosses with FTLs, *eif3x* heterozygotes were used as females and FTL; *qrt1* homozygotes as males. Some of the F1 progeny were allowed to self fertilize. Other F1 plants were crossed with the *FTL; qrt1* parent in an effort to increase the recovery of *qrt1* homozygous progeny. The F2 progeny were screened for the *FTL/+* genotype, and both *qrt1/qrt1* and *qrt1/+* were saved. These plants were also checked by PCR to confirm heterozygosity for the mutant *eif3x* allele.

Oligonucleotide primers and conditions to confirm *eif3h-1* mutant genotypes by PCR are listed in [14]. For *eif3e-1*, we combined the standard Salk-LB primer, 5′-TGG TTC ACG TAG TGG GCC ATC G-3′, with eIF3e-RP 5′- CCT CTA GAT TAT CAT GAA AGC AGT TGC CAA GTA GC-3′. Wild type *eIF3e* was detected with eIF3e-RP and eIF3e-LP 5′-GAA GAT CTA TGG AGG AAA GCA AAC AGA ACT ATG ACC TGA CGC CAC TA -3′.

Primers to confirm *LAT52:DsRed* line FTL1413 (for *eIF3e*) were 1413For 5′-GAC CAT TGC TGA CAT TGA CAT GG -3′ and 1413Rev 5′-GGC AAC TCT GTC GAG GCA CAT-3′. Primers to confirm *LAT52:YFP* line FTL567 (for *eIF3h*) were 567For 5′-TGG TCG GCC CTA AAT GTT TG-3′ and 567Rev 5′-ACC GAC ACA AGA ATC TGT GGA ACC-3′. The T-DNA left border primer was LB 5′-CAA TTC GGC GTT AAT TCA GTA C-3′. When all three primers are used together, the T-DNA insertion allele generates an ∼450 bp product and the wild-type allele gives rise to a ∼900 bp product.

### Plant growth conditions

Plants were grown in reach-in or walk-in growth chambers under fluorescent lighting with a 16 h photoperiod at 22^°^C.

### In vitro pollen germination assay

Liquid pollen germination medium contained 18% sucrose; 0.01% boric acid; 1 mM CaCl_2_; 1 mM Ca(NO_3_)_2_; 1 mM MgSO_4_ (pH adjusted to 7.0). For germination in liquid medium, dehiscent anthers were gently dabbed on the medium in a chamber slide. The released pollen grains were incubated for 6 hours to overnight. Solid germination medium (5 mM KCl, 0.01% boric acid, 5 mM CaCl_2_, 1 mM MgSO_4_, 10% sucrose, pH 7.5 with NaOH) was prepared by adding molten low melting agarose to 0.5%. The medium was dropped onto a glass slide. After solidification, pollen from dehiscent anthers was first dusted onto the medium in an attempt to minimize clumping of tetrads, before gently dabbing the anthers onto the surface. For some experiments, female pistils were also placed onto the medium in order to stimulate pollen germination and tube growth. The slides with released pollen grains were incubated upside down for 6 hours to overnight in a humid chamber made from tip boxes, wet tissue paper, and two pasteur pipettes to hold the slides. Pollen was incubated in a lighted tissue culture incubator [35].

### Alexander's viability staining [36]

A stock solution was prepared from 10 mL 95% ethanol; 1% malachite green in 95% ethanol; 5 g of phenol; 5 mL 1% acid fuchsin in water; 0.5 mL 1% orange G in water; 2 mL glacial acetic acid; 25 mL glycerol and 50 mL water (note: chloral hydrate was omitted due to restriction in use). A working solution was prepared by diluting the stock 1∶50 with water. Pollen grains were stained on a glass slide for 15 minutes and observed under the microscope.

### DAPI staining

Anthers from fresh flowers were dissected in coloration buffer (1 mg/mL DAPI (4′,6-diamino-2-phenylindole; Molecular Probes, Portland, OR, USA), in Nonidet P40 (1%); DMSO (10%); PIPES (50 mM, pH 6.9); EGTA (5 mM, pH 7.5)), incubated for 30 minutes, and observed under epifluorescent illumination.

### Microscopy

XFP fluorescence was visualized on a Zeiss Axiovert epifluorescence microscope with standard filter combinations for GFP, YFP and DsRed.

## Supporting Information

Table S1 Available FTL lines with insertion point of the fluorescent marker transgene.
(XLS)
